# Grey systems in the management of demand for palliative care services in Poland

**DOI:** 10.1186/s13561-021-00349-5

**Published:** 2022-01-05

**Authors:** Sylwia Nieszporska

**Affiliations:** Czestochowa University of Technology, Faculty of Management, Chair of Statistics and Econometrics, ul. Armii Krajowej 19B, 42-200 Czestochowa, Poland

**Keywords:** Grey systems, Demand for health services, Long-term care, Patients of palliative care facilities

## Abstract

**Background:**

The concept of care for people in a critical or even terminal health condition, who are in the last stage of their life, has become the mission of palliative care facilities. Therefore, the life of a sick patient poses a number of challenges for health care services to make sure that medical services are tailored to the trajectory of the disease, as well as the various needs, preferences and resources of patients and their families.

**Methods:**

Health systems financed from public funds need to adopt new methods of management to meet the high and arising demand for a long-term care. There are several ways of assessing the demand for long-term care services. The method recommended by the author and presented in more detail in this paper is the one relying on grey systems, which enables the estimation of forecasting models and, finally, actual forecasts of the number of potential future patients.

**Results:**

GST can be used to make predictions about the future behaviour of the system, which is why this article aims to present the possibility of using the first-order grey model *GM (1,1)* in predicting the number of patients of palliative care facilities in Poland. The analysis covers the data from 2014 to 2019, whereas the prediction of the number of patients has been additionally formulated for 2020.

**Conclusions:**

Health systems, particularly publicly funded ones, are characterised by a certain kind of incompleteness and uncertainty of data on the structure and behaviour of its individual components (e.g. potential patients or payers). The present study aims to prove how simple and effective grey systems models are in the decision-making process.

## Introduction

The concept of care for people in a critical or even terminal health condition, who are in the last stage of their life, has become the mission of palliative care facilities. According to the WHO definition, palliative care comprises all activities aimed at improving the quality of life for patients and their families coping with an illness (usually a terminal one), through the prevention and relief of suffering and treatment of pain and other physical, psychosocial and spiritual problems [1]. Such a holistic approach to the patient, incorporating nearly all aspects of living in the human environment, paradoxically paved the way to seeing death and the process of dying as a natural and inevitable stage of the life cycle, while preparing the patient and their relatives for its arrival has evolved into an important aim of palliative care facilities and hospices.

Such medical facilities provide care for patients of all ages. Their patients include children, adults and senior citizens, and yet it seems that the greatest scope of support is mostly required by the elderly, often living alone and unattended. According to international statistics [2], the percentage of people aged 60+ around the globe was 8.92% in 2018 and continues to increase year after year. It is even estimated [3] that one in five people will reach old age in 2050. Therefore, there will be a significant increase not only in the number but also in the proportion of elderly people in the world population, especially the 86+ group.

The past twentieth century, with its innovations in medicine and medical technology, changed the pattern of diseases that result in deaths today, as well as the age profile of patients at the end of life. In recent years, more people have been dying from serious chronic diseases, with deaths under the age of 60 becoming increasingly rare. Statistics in almost all countries show increasing life expectancy, longer healthy life expectancy, as well as lower infant mortality and fewer perinatal deaths. As Prentice notes [4], life expectancy in 1900 was only 31 years, while it reached a value of just over 72 years in 2018 [5]. Thus, a single century has seen an incredible leap in the number of years expected to be lived by a newly born member of the human population.

Technological changes affecting the duration and quality of human life have not been indifferent to human lifestyles in the last century. The image of contemporary senior citizens is very different from the one known many years ago, and elderly people living today often remain both physically and professionally active, thereby achieving a better and longer life in many cases.

However, as Howden-Chapman, Signal and Crane [6] point out, although there are ever more elderly people, especially those who are independent and self-reliant, and many of them may live for a very long time, yet an increasing number will need assistance over time with managing health problems as well as basic care and support in daily activities because, despite their commitment and efforts to improve their lives, biology remains relentless and takes its toll on everyone’s health. Jaul and Baron emphasise [7] that ageing is associated with physiological changes, among which the mildest ones which are nevertheless challenging for caregivers of elderly people include: reduced visual and auditory acuity, slower reaction times and impaired balance.

It would seem that the simplest way of caring for an elderly person is to provide family-based support. Recently, however, there has been a widespread and increased degree of migration and emigration, driven by the quest for a better life, well-paid work or better social security among young people. For the elderly, according to Silverstein and Giarrusso [8], this means that the modern Western civilisation no longer has room for living in multi-generational homes and families, or even for maintaining strong and close family relations. The increased professional activity of women on the labour market is also of relevance for the situation of senior citizens and their place in society. As highlighted by World Bank reports [9], it is women who constitute the pillar of informal care for the elderly, and involvement in such care is incompatible with full-time occupational activity. The feminisation of the labour market also has an important, although not direct, consequence for senior citizens: women give birth to fewer children, thus leading to a lack of replacement of generations, and a deficit of family carers for the elderly.

Therefore, not only the dynamically changing age structure of the world’s population, but also changes in the lifestyle, nature and place of work, as well as cultural changes have resulted in numerous challenges for the policies and economies of many countries, necessitating the adaptation of the labour market, pension and social security systems and, above all, the health and medical care systems to the new conditions.

The World Health Organisation has alerted [10] that health policies in many countries have not kept pace with the health needs of the elderly, the supply of services is not in balance with demand, and existing institutional support leaves much to be desired in terms of the quality of care that people receive at the end of life. As experts have stressed, *it is essential that services for older people are included in universal health care packages. At the same time there needs to be good coordination between the health and social services to provide optimal care when needed. The new package of tools supports healthy ageing with a person-centred and coordinated model of care* [11]*.*

Therefore, the life of a sick elderly patient poses a number of challenges for health care services to make sure that medical services are tailored to the trajectory of the disease, as well as the various needs, preferences and resources of patients and their families. Such care also calls for partnership and cooperation between different groups and many settings. This raises a number of issues of a sociological, medical and psychological nature, as well as those related to strategy, planning and even policies [12], with the assessment of potential demand for services such as long-term care being among the most essential issues.

## Methods

### Demand for long-term care

Demand is the essential phenomenon that determines the market, including the market for health care services. According to economic theory, demand is the *inverse relationship between the price of a good and the quantity that consumers are willing and able to purchase in a given period of time, assuming that all characteristics of the market situation remain unchanged* [13]. Demand and its size are determined by a number of variables. These include: price, level of real income of the consumer group, buyers’ preferences and tastes, prices of substitute and complementary goods and services available on the market, the number and structure of the population, expectations connected with changes in prices and income. In addition, demand is influenced by characteristics such as education, age, gender, religious beliefs as well as other qualitative factors and variables.

In the case of demand in the market for medical services, one speaks of demand for health as well as demand for a medical service.

In the former approach, health is viewed as capital of high value, but also as a prerequisite for any human activity [14]. While individuals determine their own health, it is also influenced by variables such as genetic predispositions, environmental factors as well as unpredictable, random events which can have a significant positive or negative impact on individuals’ efforts to maintain and improve their health. However, humans are the main ‘producers’ of their own health, and the production of health itself can be interpreted as a consequence of the consumption of capital that individuals will accumulate in the course of their lives, leading a specific lifestyle.

The contribution and effort made by individuals who seek to maximise utility^1^ and to manage their health capital in an optimum way over the life cycle can therefore be seen as a kind of investment in health.

In the context of this approach, the demand for health is represented using the Grossman model based on the theory of optimising the individual’s utility over time [16]. The individual is treated as a consumer of health, but also as an investor in their health capital.

Apart from individuals, the second source of health expenditure, health investment and support for health capital includes the health care providers and their services. However, there is a theory [17] whereby the relationship between the capital accumulated by the individual and health services is negative.

Patients and their behaviours have an impact on the demand for health as well as for medical services. The latter depends on the patients’ education, wealth, age and health capital accumulated over the years, but it is also influenced by wage rates and the prices of these services.

Analyses of the demand for long-term care, as for any other medical care services, do not liken the demand only with the need to receive such care, but also take into account the patient’s ability and willingness to purchase such a service and pay for it. According to Suchecka [18], important factors influencing the demand for health care services also include the increasing morbidity, progress in medical sciences, cultural factors or the health care financing system. In view of the latter variable, it is important that analyses of demand for formal long-term care should also consider the supply of informal care services, the availability and prices of privately funded care, the income and even the assets of beneficiaries [19].

As a consequence of this approach, there is a very meticulous view of the demand for long-term care services, where a distinction is made between different types of services, as well as the types of institution to provide the service. Some patients may have a demand for psychiatric services while others are fully dependent and require round-the-clock nursing care. The needs of some patients can be met by a publicly funded formal support institution while others rely on services provided by formal commercial institutions, inextricably linked to financial costs borne directly by the elderly or their families, and yet other patients benefit from informal care provided by their loved ones or by social assistance institutions guaranteed by the central or local government.

Demand for long-term care is a function of a number of variables. These include: age, gender, marital status, dependency level of the elderly, physical health status, mental health status, income, patient preferences, cost of care [19].

According to the report by Wren, O’Reilly et al .[20] the following breakdowns are needed to assess demand: the number of elderly people by age and gender, household composition, marital status, socio-economic group, severity of disability/disease as well as the supply of such services.

The Irish report from a study into the demand for long-term care for the elderly indicates that the degree of disability is associated with the main medical condition among potential residents of long-term care homes, the most common ones being dementia, stroke and mobility problems, resulting in frequent falls [20]. Moreover, it was noted that increasing longevity increases the demand for long-term care. Proximity to death increases the likelihood of using nursing homes and formal in-home care, but reduces the availability of informal support. When analysing the expenditure on institutional care and home care in the Netherlands, de Meijer et al. [21] found that expenditure on long-term care for people who lived alone and later died of diabetes, mental illness, stroke, respiratory or gastrointestinal disease was higher than expenditure incurred on services for people who died of cancer.

According to studies by Wren et al. [20], women are more in demand for long-term care than men. Likewise, people living alone have a higher demand as opposed to those living with a spouse and/or children. Increased demand is also observed among elderly people whose immediate family includes women working full-time rather than only part-time. Indeed, women with lower labour market involvement are more involved in childcare and informal care of the elderly.

People with greater socio-economic resources are more predisposed to pay for additional home care, thus forgoing the use of institutional forms of long-term care.

As regards the variables which determine demand, Worall divides them [22] into macro- and microeconomic ones. The former include:

- prevalence of diseases,
- mortality rates,
- cultural background to elderly care,
- future health competences,
- criteria for qualifying patients for government-funded care,
- availability of free long-term care services,
- future patterns of care,
- health improvement indicators.

Microeconomic factors include:

- estimated survival time,
- type and number of diagnoses given,
- level of disability,
- marital status,
- living status,
- the degree of family support,
- personal control,
- informal care,
- previous stays in hospital or care homes,
- number of medications taken,
- age,
- gender,
- education,
- the presence of depressive symptoms,
- and others.

However, a slightly different approach is beginning to prevail in contemporary trends in the analysis of the long-term care market, i.e. public spending is seen as substitutive versus private spending on long-term care, which results in distortions in the analysis of the demand for this type of care. Thus, public spending does not fully reflect the outlays incurred for long-term care and is often associated with costs indirectly borne by the patients themselves. Moreover, Łuczak links their amount to the consequences of political decisions and resolutions which seem to have no correlation with the actual needs of ageing societies [23]. Considering all these factors, the division of long-term care financing into public and private sources becomes ambiguous and insufficient. Therefore, the proposal formulated by Costa-Font, Courbage and Swartz [24] is based on the division of long-term care financing mechanisms according to the fund accumulation period:

- *ex ante* and
- *ex post*.

The former one concentrates on how such benefits are funded prior to the onset of disability and includes the following variables:

- insurance (social or private),
- savings,
- preventive measures aimed at minimising dependence and thus reducing the costs resulting from problems with independent living.

*Ex-post* instruments include all care funding arrangements implemented and launched following the onset of disability. They include:

- subsidising and supporting formal and/or informal care,
- family support,
- use of assets such as real estate.

In contrast to the classic approach, the one presented above takes into account the mixed, public-private nature of financing, it emphasises the importance of prevention and preventive health care in the creation of long-term care costs, which, according to Eling and Ghavibazoo [25], makes *ex ante* and *ex post* mechanisms common in analyses of the long-term care market in many developed economies.

### Methods for measuring and estimating demand

The modelling of selected sectors, branches and components of the national economy is the domain of the so-called macromodels, which are helpful not only for in-depth macroeconomic analysis, but also for economic forecasting [26]. Macromodels take into account not only the relationship between sectors and mutual exchange between those sectors, but also the role of government, exchange with other countries, as well as fiscal and monetary policy of the country concerned [27].

Macromodels are constructed on the basis of econometric models, i.e. those whose *parameters (...) are estimated on the basis of statistical data, using appropriate estimation methods* [26], as well as simulation models, based on econometric models but using, for example, non-observable variables (including demand and supply).

In demand modelling, modern econometrics distinguishes between four essential approaches [28]. The first one focuses on modelling demand for individual consumer goods or services using single-equation econometric models. The second approach involves the construction of complete demand models and covers the entire demand structure using a system of demand functions. The third approach relies on econometric macromodels for the whole economy, most often expressed by multiple-equation models. The fourth class of models includes those that describe the key elements of the market and most commonly involve the analysis of local markets.

Nowadays, micro-simulation and macro-simulation models are becoming instrumental in solving the problem of modelling demand for long-term care services*.*

Micro-simulation models rely on representative samples to simulate changes in disability levels and long-term care use as well as expenditure. However, these models require regular surveys of the same large sample of people and, consequently, high investments in terms of time, money and personnel.

Another concept for modelling the demand for long-term care [22] focuses on a mathematical formula aimed at finding the optimal allocation of financial resources (contracts with providers), taking into account and determining estimates of patient demand for this type of service and discounts for providers arising from specific volume or time commitments. This model can therefore be used to obtain forecasts of the generated long-term care cost savings, also at the local level.

One of such models is based on the theory of grey systems, i.e. systems where information on boundaries, internal structure, interaction with the surroundings is incomplete and sometimes even uncertain [29]. In accordance with the general systems theory, widely described in Poland as early as in the 1960s by Mazur [30], it is assumed that each system has a certain number of inputs and outputs, i.e. factors that affect the system but also determine other systems by penetrating into the surroundings.

The simplest case of a model based on THE grey systems theory GM (1,1) is commonly used for forecasting. GM (1,1) defines a grey forecasting model with one input variable. The initial sequence of observations is represented by the vector:

1 $$ {X}^0=\left\{{X}^0(1),{X}^0(2),\dots, {X}^0(n)\right\}, $$

where *X*^0^(*k*) > 0 and *n* is the number of observations.

The observations collected into vector (1) are then accumulated to generate a monotonically increasing sequence, forming a new vector of observations:

2 $$ {X}^1=\left\{{\Sigma}_{k=1}^1{X}^0(k),{\Sigma}_{k=1}^2{X}^0(k),\dots, {\Sigma}_{k=1}^n{X}^0(k)\right\}=\left\{{X}^1(1),{X}^1(2),\dots, {X}^1(n)\right\}, $$

where *X*^0^(1) = *X*^1^(1).

Now, let vector ***X***^*1*^ be a function of time *t* and be a solution to the following first order differential equation:

3 $$ \frac{dX^1(t)}{dt}+{aX}^1(t)=b. $$

In Eq. (3), *a* is the so-called *development coefficient* and *b* is the *forcing coefficient* [29]. These coefficients are calculated from the following formula:

4 $$ \left[\begin{array}{c}a\\ {}b\end{array}\right]={\left({B}^TB\right)}^{-1}{B}^TY, $$

where: $$ B=\left[\begin{array}{c}\begin{array}{cc}\frac{-1}{2}\left[{X}^1(1)+{X}^1(2)\right]& 1\\ {}\frac{-1}{2}\left[{X}^1(2)+{X}^1(3)\right]& 1\\ {}\dots & \dots \end{array}\\ {}\frac{-1}{2}\left[{X}^1\left(n-1\right)+{X}^1(n)\right]\kern0.5em 1\kern1em \end{array}\right] $$, while $$ Y=\left[\begin{array}{c}\begin{array}{c}{X}^0(2)\\ {}{X}^0(3)\\ {}\dots \end{array}\\ {}{X}^0(n)\end{array}\right] $$.

The solution of Eq. (3) for a unit leap of variable *t = 1* is an estimated value of vector *X*^*1*^ of the form:

5 $$ {\hat{X}}^1\left(t+1\right)=\left({X}^0(1)-\frac{b}{a}\right){e}^{- at}+\frac{b}{a} $$

In order to find the predicted value of the original sequence, inverse cumulative generation is applied, resulting in the following:

6 $$ {\hat{X}}^0\left(t+1\right)={\hat{X}}^1\left(t+1\right)-{\hat{X}}^1(t). $$

Finally, the forecast for t = 2, 3, ..., n for the GM (1,1) model has the following form:

7 $$ {\hat{X}}^0\left(t+1\right)=\left[{X}^0(1)-\frac{b}{a}\right]\left({e}^{- at}-{e}^{-a\left(t-1\right)}\right) $$

with the forecast error vector given in this formula:

8 $$ \varepsilon ={X}^0(t)-{\hat{X}}^0(t). $$

For the purposes of long-term care forecasting, one of the most important sequences to predict is the number of patients who require such care in the future and the associated cost. However, modelling the demand for long-term care services using grey system models poses many problems, mainly due to the complex nature of long-term care systems. This is because there is no single treatment or service that patients could use and that practitioners could refer to in order to model such long-term care. Secondly, the illnesses associated with long-term patients often represent a whole set of health conditions and might include both mental and physical disabilities. Thirdly, data on social care services and informal care provided to long-term care patients are often difficult to obtain and are linked to other types of health services the patient may have received. Fourthly, long-term care is subject to multiple reforms. Given all these considerations, it is not obvious whether the historical data needed for modelling based on grey systems theory are sufficient to make reliable predictions for the future. However, the undoubted advantage of using grey systems models is that the method is straightforward and transparent and, above all, it does not require costly and time-consuming research, which means that the method based on such models is extremely useful.

## Results

### Grey systems modelling in the assessment of demand for hospice services in Poland

In order to present grey modelling and its usefulness for estimating demand for long-term care, an attempt was made to forecast only the realised demand for hospice services in Poland, which is due to the lack of public data on, e.g. the number of patients waiting for such services. Data on the number of patients were collected for 2014–2019, covering 16 provinces (voivodships) of Poland, each of which has its separate branch of the National Health Fund.

Demand modelling involved the use of the *GM (1,1)* grey system to estimate the equations forecasting the patient numbers for the aforementioned 16 regions and, consequently, to provide forecasts for 2015–2020.

The forecast of the number of hospice patients derived from the grey systems modelling does not take into account any variables other than time. A sequence of six observations within each province was therefore used to determine the forecast values and relative *ex post* errors, starting from the second realisation (*t = 2*) onwards^2^, up to the sixth one. For the year 2020, only the forecast values were determined without giving their actual equivalents, which is due to the lack of access to such data at the time when the present study was prepared.

Bearing in mind the forecasting algorithm presented and discussed above, the first step was to determine the vector of actual values of the number of hospice patients in 2014–2019 (2014: *t = 1*, 2015: *t = 2,* 2016: *t = 3,* 2017: *t = 4,* 2018: *t = 5,* 2019: *t = 6*) for each province separately, based on the collated database.

After calculating the initial partial sums, the following vector was obtained in the next step:

$$ {X}^1=\left\{{X}^1(1),{X}^1(2),{X}^1(3),{X}^1(4),{X}^1(5),{X}^1(6)\right\}, $$

and on this basis the ***B*** matrix and the ***Y*** vector were determined to help in determining the forecast equations (Table 1).

**Table 1:** Forecasting equations for patient numbers

Province	Forecast equation
DOLNOŚLĄSKIE	${\hat{X}}^0\left(t+1\right)=\left[{X}^0(1)-\frac{7245.324}{-0.046}\right]\left({e}^{0.046t}-{e}^{0.046\left(t-1\right)}\right)$
KUJAWSKO-POMORSKIE	${\hat{X}}^0\left(t+1\right)=\left[{X}^0(1)-\frac{6284.629}{-0.010}\right]\left({e}^{0.010t}-{e}^{0.010\left(t-1\right)}\right)$
LUBELSKIE	${\hat{X}}^0\left(t+1\right)=\left[{X}^0(1)-\frac{3234.193}{-0.047}\right]\left({e}^{0.047t}-{e}^{0.047\left(t-1\right)}\right)$
LUBUSKIE	${\hat{X}}^0\left(t+1\right)=\left[{X}^0(1)-\frac{2737.329}{-0.016}\right]\left({e}^{0.016t}-{e}^{\left(0.016\Big(t-1\right)}\right)$
ŁÓDZKIE	${\hat{X}}^0\left(t+1\right)=\left[{X}^0(1)-\frac{5600.454}{-0.014}\right]\left({e}^{0.014t}-{e}^{0.014\left(t-1\right)}\right)$
MAŁOPOLSKIE	${\hat{X}}^0\left(t+1\right)=\left[{X}^0(1)-\frac{6061.948}{-0.042}\right]\left({e}^{0.042t}-{e}^{0.042\left(t-1\right)}\right)$
MAZOWIECKIE	${\hat{X}}^0\left(t+1\right)=\left[{X}^0(1)-\frac{8843.804}{-0.0555}\right]\left({e}^{0.055t}-{e}^{0.0555\left(t-1\right)}\right)$
OPOLSKIE	${\hat{X}}^0\left(t+1\right)=\left[{X}^0(1)-\frac{2193.076}{-0.0514}\right]\left({e}^{0.0514t}-{e}^{0.0514\left(t-1\right)}\right)$
PODKARPACKIE	${\hat{X}}^0\left(t+1\right)=\left[{X}^0(1)-\frac{4066.862}{-0.047}\right]\left({e}^{0.047t}-{e}^{0.047\left(t-1\right)}\right)$
PODLASKIE	${\hat{X}}^0\left(t+1\right)=\left[{X}^0(1)-\frac{2206.915}{-0.019}\right]\left({e}^{0.019t}-{e}^{0.019\left(t-1\right)}\right)$
POMORSKIE	${\hat{X}}^0\left(t+1\right)=\left[{X}^0(1)-\frac{5267.882}{-0.024}\right]\left({e}^{0.024t}-{e}^{0.024\left(t-1\right)}\right)$
ŚLĄSKIE	${\hat{X}}^0\left(t+1\right)=\left[{X}^0(1)-\frac{11469.032}{-0.022}\right]\left({e}^{0.022t}-{e}^{0.022\left(t-1\right)}\right)$
ŚWIĘTOKRZYSKIE	${\hat{X}}^0\left(t+1\right)=\left[{X}^0(1)-\frac{3144.951}{-0.031}\right]\left({e}^{0.031t}-{e}^{0.031\left(t-1\right)}\right)$
WARMIŃSKO-MAZURSKIE	${\hat{X}}^0\left(t+1\right)=\left[{X}^0(1)-\frac{4031.232}{0.015}\right]\left({e}^{-0.015t}-{e}^{-0.015\left(t-1\right)}\right)$
WIELKOPOLSKIE	${\hat{X}}^0\left(t+1\right)=\left[{X}^0(1)-\frac{8019.070}{-0.006}\right]\left({e}^{0.006t}-{e}^{\mathrm{0..006}\left(t-1\right)}\right)$
ZACHODNIOPOMORSKIE	${\hat{X}}^0\left(t+1\right)=\left[{X}^0(1)-\frac{3296.178}{0.005}\right]\left({e}^{-0.005t}-{e}^{-0.005\left(t-1\right)}\right)$

Source: Author’s own calculations

In the case of most provinces, the estimated parameter *a* of vector $$ \left[\begin{array}{c}a\\ {}b\end{array}\right] $$ 3 takes on negative values. The value of this parameter is positive only for the Warmińsko-Mazurskie and Zachodniopomorskie provinces, which means that, according to the estimates, precisely these provinces saw an annual increase in the number of patients of the analysed institutions in 2014–2019. Actual values suggest something completely different, namely they reveal an average annual increase in the number of patients of the facilities under study in almost all provinces except for Zachodniopomorskie. Such a large discrepancy with the estimated values implies the need to examine the accuracy of the forecasts based on the equations estimated above.

## Discussion

At the turn of the twenty-first century, palliative care services, notably hospices, changed their *modus operandi* and approach to patients. In the 1970s, patients were divided into those who were healthy, those who were sick and, in the next step, those who were cured and those who did not survive.

Source: Lynn J, Adamson DM (2003) Living Well at the End of Life Adapting Health Care to Serious Chronic Illness in Old Age. RAND: 6–7. p.7.

**Fig. 1:**
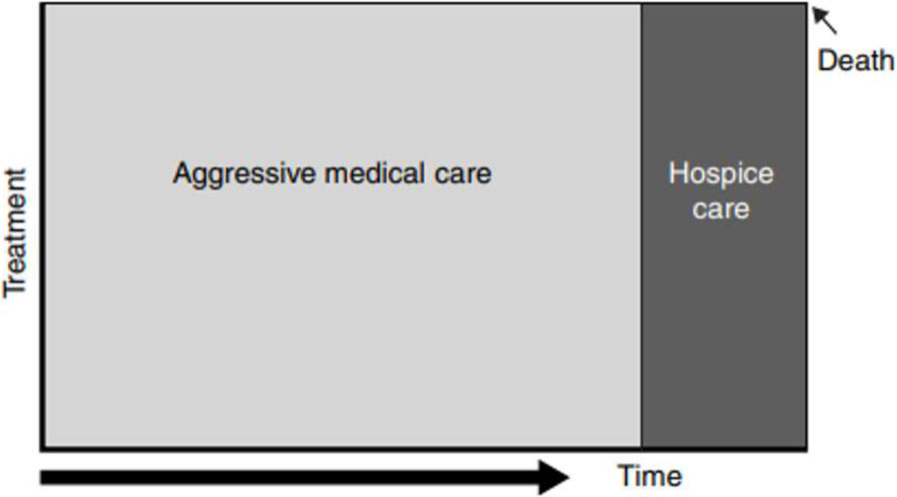
Traditional palliative care model. Source: *Lynn J, Adamson DM (2003) Living Well at the End of Life Adapting Health Care to Serious Chronic Illness in Old Age. RAND: 6-7. p.7*

The death was attributed to wrong medical treatment, which led to the conclusion that a different therapy was needed [32] (Fig.1). Medical interventions remained highly invasive, and the patients themselves quickly changed their status from ‘ill’ to ‘terminally ill’.

**Fig. 2:**
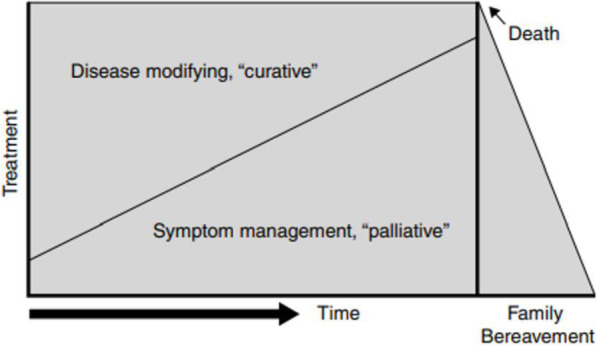
Contemporary palliative care model. Source: Lynn J., Adamson D.M., *Living Well at the End of Life: Adapting Health Care to Serious Chronic Illness in Old Age*, op.cit., p. 7

The contemporary approach to palliative care assumes, in accordance with medical knowledge, that the fate of the patient is not determined as the patient may either live with the illness for many years, or die. Therefore, palliative care today focuses not so much on organising care only to ensure a dignified death for the patient, but it focuses primarily on living (Fig. 2). It is assumed that patients can live a long life despite their illness, with periods of disability, infirmity and exacerbation of symptoms, each of which may prove fatal.

While palliative care is largely provided for cancer patients, the WHO notes that patients with other life-threatening conditions, suffering during end-of-life treatment, are also included in such care [1]. Therefore, potential patients of palliative care facilities are those suffering from the following:

- Alzheimer’s disease and other dementias,
- cardiovascular diseases (excluding sudden death),
- cirrhosis,
- chronic obstructive pulmonary disease,
- diabetes,
- HIV/AIDS,
- kidney failure,
- multiple sclerosis,
- Parkinson’s disease,
- rheumatoid arthritis,
- drug-resistant tuberculosis (TB).

In Poland, palliative care under general insurance is provided for patients with the following conditions [33]:

- disease caused by the human immunodeficiency virus (HIV),
- cancer,
- consequences of inflammatory diseases of the central nervous system,
- systemic primary atrophy involving the central nervous system,
- cardiomyopathy,
- respiratory insufficiency not elsewhere classified,
- decubitus ulceration.

However, diseases which are generally accepted as ones that qualify patients for palliative care, both worldwide and in Poland include, in particular, oncological, cardiac and psychiatric conditions. Each of these types of diseases proceeds differently in palliative patients, which determines the priorities of such care, its planning and even the cost management in providing such services.

**Fig. 3:**
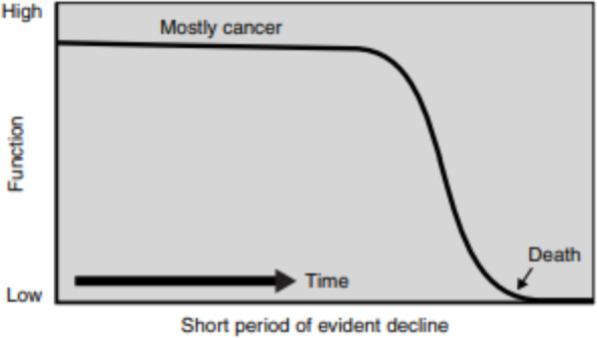
Model of a trajectory of an illness due to cancer. Source: *Better Palliative Care for Older People (2004*). Davies E, Higginson IJ (ed.), World Health Organization. p.15

The course of oncological diseases is highly individual and depends on multiple factors. The most common of these include the stage of the disease and the patient’s reaction to radio- or chemotherapy. Many patients remain active for a long time until their body no longer responds to treatment. Patients have been observed to display a short period of rapid decrease in activity, consequently leading to rapid death (Fig. 3).

Cancer patients are willing to get involved in treatment planning, they analyse and need relevant information and are relatively well adapted to social life, as long as they receive palliative care from the moment they were diagnosed [34].

**Fig. 4:**
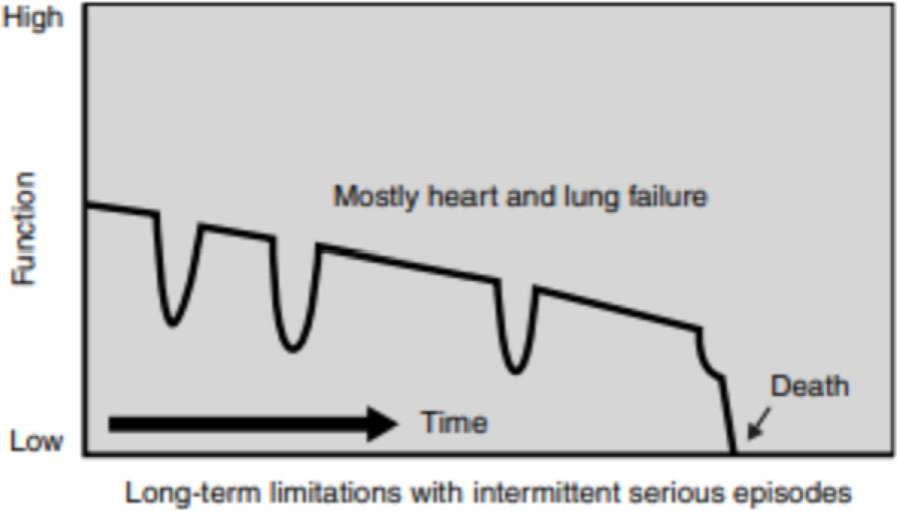
Model of an illness trajectory for heart and lung diseases. Source: *Better Palliative Care for Older People*, op.cit., p.16

The care of cardiac patients takes a somewhat different trajectory. They are most commonly affected by fairly regular attacks of dyspnoea, pain and resultant anxiety. The process of passing away is usually gradual or it may occur suddenly during a crisis (Fig. 4). Cardiac patients and their families often find it difficult to understand and accept treatment and, although they have no clear knowledge of their condition, they avoid talking openly about it.

**Fig. 5:**
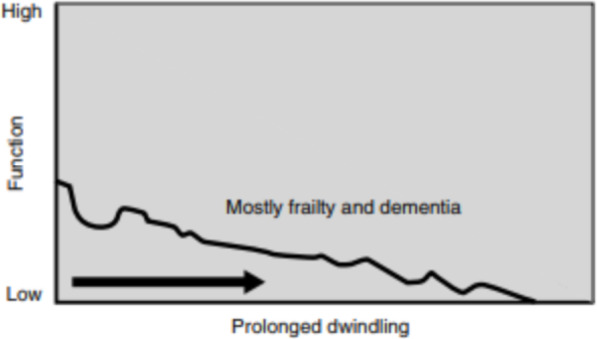
Model of an illness trajectory for dementia. Source *Better Palliative Care for Older People*, op.cit., p. 16

Among psychiatric patients receiving palliative care, the loss of contact and emotional bonds with family and carers is most commonly reported, resulting in a lack of awareness of patients’ needs and wishes. Such patients suffer from a progressive deterioration of consciousness and capabilities throughout their illness [34] (Fig. 5). The median of survival period for dementia patients between diagnosis and death is eight years.

One of the major issues that palliative care focuses on is the presence of not only a specific disease, but also the related pain and suffering. The severity of pain and its prevalence has a profound impact on the planning of patient care programmes, the timing of services, the nature and magnitude of needs. According to the WHO recommendation, the prevalence of pain for each disease category is a variable that influences the assessment of palliative care needs and it is combined with mortality data related to diseases that require palliative care^4^.

There are many methods for identifying and classifying pain^5^. One of such methods, described by Higginson, classifies the aforementioned prevalence of pain in adults with diseases qualifying patients for palliative care as follows [1]:

- patients with cancer – 84%
- with Alzheimer’s disease and other dementias – 47%,
- with cardiovascular diseases (excluding sudden death) – 67%,
- with cirrhosis – 34%,
- with chronic obstructive pulmonary diseases – 67%,
- with diabetes – 64%,
- with HIV/AIDS – 80%,
- with renal insufficiency – 50%,
- with multiple sclerosis – 43%,
- with Parkinson’s disease – 82%,
- with rheumatoid arthritis – 89%,
- with drug-resistant tuberculosis (TB) – 90%.

This percentage is multiplied by the number of deaths (both variables for each category of listed diseases, respectively) to obtain the estimated number of patients that require palliative care.

The overarching goal of all health care systems, regardless of the country, is to ensure the health of the society and to meet the needs of its members through the provision of medical services.

Depending on their form and structures, and the perceived role of government institutions, health systems are generally subdivided into several basic types.

One of them is the type of health care model initiated in the nineteenth century in Germany, known today as the social health insurance model or the Bismarck model [36]. In this model, all social groups are covered by universal health insurance, and the role of insurance bodies is played by insurance funds specifically set up for this purpose (i.e. the sickness funds). They collect contributions from employers, employees and general tax revenues as the main source of funding for health care [37].

In modern-day Germany, there is also the possibility of taking out additional private health insurance.

Each of the multiple health insurance funds insures its members against loss of independence, thus granting long-term care benefits. For privately insured individuals, a special insurance contract is required to cover the loss of ability to live independently [38].

Long-term care and its extent in Germany does not depend on age, income or the number of years spent working. The degree of needs is the only criterion for the provision of such care. Long-term care is granted by the health insurance services only to people who, as a result of a physical, mental or intellectual disability or illness, require assistance with daily activities for at least 6 months.

Insurance against the loss of the ability to live independently covers the following:

- provision of home care and home assistance by outpatient care centres or individual carers,
- a flat-rate allowance to cover the full costs of care in a nursing home,

and the amounts of those benefits, which remain in compliance with relevant legislation, are determined by the degree of dependence on third parties.

The social health insurance model exists in Germany, Austria, France, Belgium, the Netherlands, Luxembourg, and it existed in Poland during the interwar period and in 1999–2003.

Another type of health care model found in Europe is the budget model, also called the national health service model or the Beveridge model [18].

The National Health Service in the United Kingdom provides health care for all citizens through the tax system. It is also funded from insurance premiums and user fees [39]. The central budget allocates and legally regulates the sums allocated to health care financing, thus making the health care system dependent on and controlled by public administration. Thus, the government is the main supervisor and provider of services, implementing the centrally defined health policies and formulating strategies for the entire system.

The British system focuses on fulfilling citizens’ rights and the provision of medical care to every citizen regardless of their ability to pay. However, this protection covers only a fixed ‘basket’ of guaranteed services. All services going beyond this basket must be financed from voluntary supplementary insurance.

Within the basket of guaranteed services and long-term care services, British nationals are entitled only to nursing care, whereas other necessary costs of such care are borne by the patient or the local authority (if the patient is eligible for their support). Local authorities are most likely to help people with the lowest financial resources in this respect by taking over all their private pensions, as well as most of the social security benefits the person is entitled to [40].

In addition to the UK, the national health service model operates in Ireland, Iceland, Sweden, Norway, Finland and Denmark.

The Polish health care system includes features of both the German and the British model. Since 2003, all Polish citizens have been covered by universal and compulsory insurance at the National Health Fund (NFZ) [41], which is a central authority. Under this insurance, Polish patients receive a bundle of guaranteed health services.

As regards in-patient long-term care, Poland has nursing-and-therapeutic facilities as well as nursing-and-care facilities with either a general or a psychiatric profile, whereas palliative care available to everyone is mostly provided by hospices.

Under Polish law, the choice of the relevant long-term care treatment provider depends on the patient’s degree of independence.

According to Statistics Poland data, there were 106 hospices in Poland in 2018 (Table 2), with an upward trend in their number over several years.

**Table 2:** Hospices in Poland

	2015	2016	2017	2018
Number of hospices	82	80	95	106
Number of in-patients (‘000 persons)	18.0	18.4	19.0	20.6
number of beds (‘000)	1.6	1.6	1.8	3.4
Average duration of stay (days)	25.4	27.1	30.7	30.0

Source: Author’s own analysis based on data from the Centre of Health Information Systems and the Statistics Poland (GUS)

**Fig. 6:**
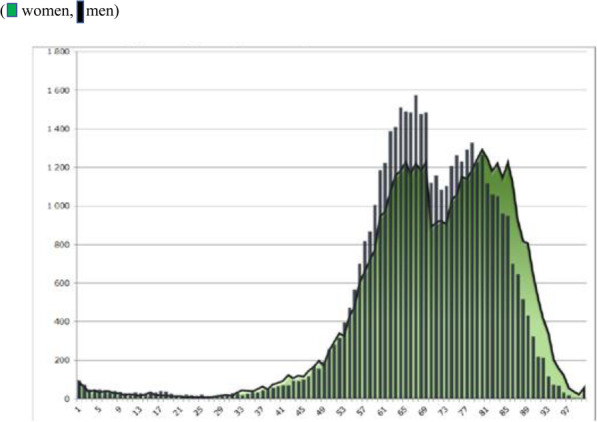
Number of patients in palliative care facilities in Poland in 2015, by age (women, men). Source: *Sprawozdanie z działalności Narodowego Funduszu Zdrowia za 2015 rok* [*Report on the activities of the National Health Fund for 2015*], National Health Fund, Warsaw, June 2016, p. 95 [43]

In all types of long-term care facilities, i.e. nursing-and-therapeutic facilities, nursing-and-care facilities, hospices and palliative care units, more than 78% of patients are over 65 years of age [42]. In 2015, the age threshold which marks a significant increase in the number of patients is 50 years of age (Fig. 6). Approximately equal numbers of women and men use hospice services. Significant gender differences are observed between the ages of 60 and 75, with men outnumbering women.

These sample data on the number of long-term care patients justify the conclusion that the demand for such services is constantly increasing, which entails, especially for publicly funded health care systems, the need to monitor such demand and, above all, to anticipate it.

The idea to calculate a demand forecast makes sense if the error of this forecast is the smallest. The order of the value of the estimated number of patients is closest to the actual value for Mazowieckie, and its mean *ex post* forecast error ((${x}^0-\hat{x^0\Big)/}{x}^0\Big)$ for 2015–2019 amounted to − 0.001% (Table 3).

**Table 3:** Relative forecast errors for the number of hospice patients

Province	Relative forecast error				
	2015	2016	2017	2018	2019
**Dolnośląskie**	0.0051	0.0019	−0.0114	0.0020	0.0034
**Kujawsko-Pomorskie**	−0.0036	−0.0003	0.0113	−0.0079	0.0004
**Lubelskie**	0.0073	−0.0166	0.0031	0.0144	−0.0078
**Lubuskie**	0.0015	−0.0029	0.0006	0.0016	−0.0007
**Łódzkie**	0.0108	−0.0294	0.0119	0.0173	−0.0121
**Małopolskie**	0.0211	−0.0368	0.0061	0.0131	−0.0045
**Mazowieckie**	0.0336	−0.0268	−0.0257	0.0104	0.0084
**Opolskie**	−0.0032	−0.0271	0.0221	0.0334	−0.0280
**Podkarpackie**	−0.0367	0.0105	0.0247	0.0340	−0.0375
**Podlaskie**	0.0146	−0.0338	0.0146	0.0098	−0.0067
**Pomorskie**	0.0212	0.0085	0.0081	0.0199	0.0149
**Śląskie**	−0.0176	0.0071	0.0172	0.0077	−0.0153
**Świętokrzyskie**	−0.0102	0.0095	−0.0023	0.0131	−0.0102
**Warmińsko-Mazurskie**	−0.0059	0.0160	0.0061	−0.0400	0.0216
**Wielkopolskie**	0.0124	−0.0081	−0.0167	0.0073	0.0045
**Zachodniopomorskie**	−0.0241	0.0328	−0.0055	0.0043	−0.0093
**Average**	**0.001634**	**−0.00597**	**0.004009**	**0.00877**	**−0.00492**

Source: Author’s own calculations

Given the small number of time units, one cannot expect that the rest of the grey systems model (${x}^0-\hat{x^0}\Big)$ fulfill the assumptions of the Classical Least Squares Method, used to estimate vector $$ \left[\begin{array}{c}a\\ {}b\end{array}\right]. $$ The essence of the grey model *GM (1,1)* is such a goodness of fit of the theoretical values to the real ones that, measured by the value of the *ex post* error*,* will be acceptable. This means that the forecasts will become acceptable with the accepted *ex post* error.

## Conclusions

The management of demand or, rather, such management of the health care system that will ensure the fulfilment of patients’ needs, is a serious challenge faced by systems financed mainly from public funds. On the one hand, especially considering the market aspects, high demand for a specific service entails the need to adapt the organisational structure of the system and the range of services provided by facilities, to prepare medical staff to the changing conditions on the part of beneficiaries. On the other hand, financial resources, which are usually limited, imply such an allocation and redistribution which will not as much ensure full cost-effectiveness of individual facilities but will enable the provision of services from a given guaranteed ‘basket’.

However, regardless of the problems arising from the changing medical needs in the society, planning and system management continue to be based on market surveys, notably from the demand side.

As shown above, there are several ways of assessing the demand for long-term care services. The method recommended by the author and presented in more detail in this paper is the one relying on grey systems, which enables the estimation of forecasting models and, finally, actual forecasts of the number of potential future patients.

The method used by the author was affected by some error already at the stage when the database was prepared, due to the unavailability of information on the number of patients waiting for admission to the facilities concerned. The data on the number of patients waiting for admission seem to be very important for determining a reliable and credible demand forecast for hospice services, and the estimated demand for hospice services, modelled and projected on the basis of grey systems, should be understood as realised demand.

The database collected by the author is not perfect, not only because of the unavailability of information on unrealised demand, but also because of the length of statistical series. The publicly accessible information used by the author, in particular the data available from the National Health Fund in Poland, leaves much to be desired in terms of updates. Indeed, the reports become available on the website with a very significant time lag, which consequently limited the database created by the author to 2014–2019 and, thus, limited the forecast period to the year 2020.

Ultimately, however, modelling with the GM (1, 1) model provides a conducive environment for forecasting the number of hospice patients. Not only do grey models seem to be much simpler to use than other econometric models in the context of calculations or data collection, but the conducted research on the use of grey models [44] gives a reason to believe that their acceptability measured, e.g., by the forecasting error from the *ex post* group remains high in the short forecasting horizon as well. Nevertheless, this does not change the fact that one of the drawbacks of this model continues to be the failure to fulfil the assumptions related to the random component of the model (i.e. the size of ${x}^0-\hat{x^0}\Big),$ or, more precisely, the assumptions of the Classical Least Squares Method used to determine the vector of parameters used in the forecast equations.

Another persisting drawback of grey modelling is the rejection of the need to include any variables other than the number of patients.

Some doubts may also be raised by the length of time series used for estimation. The observation period covered six years for each Polish province. On the other hand, if the observation period were to be extended, this could necessitate the use of a different class of grey model (e.g. a rolling grey model with a short forecasting window), which would consequently make it more complicated and more difficult to implement, but, on the other hand, it could provide a starting point for further research in this area.

However, regardless of the author’s concerns about the reliability of the presented models and forecasts derived from them, it is undeniable that these models are an efficient and useful tool for assessing the demand for services offered by long-term care facilities. Their role seems to be all the more important if we highlight the diversity of regions in terms of supply and demand for such services.

It is important to emphasise the fact and the possibility of using grey systems modelling to estimate the number of patients of such healthcare facilities in future. This aspect seems to be extremely important from an economic perspective since it allows the estimated number of patients to be used to assess future costs of long-term care. In the presented paper, the data about hospices’ patients in general (adult and children) were used to estimate a model. If the data about only adult patients is used, it will be easy to assess the future needs of senior citizens in the realm of long-term care and related adjustments to the health care market.

## Data Availability

The datasets generated during and/or analysed during the current study are available in the www.nfz.gov.pl.

## Footnotes

1 For more on the concept of utility in health care, see, e.g. [15].

2 Therefore, it was assumed that the first theoretical realisation is always an ideal forecast with an error of zero.

3 Therefore, it was assumed that the first theoretical realisation is always an ideal forecast with an error of zero. 3 *a* is the growth exponent, while b is the control variable of the grey model.

4 The WHO also carries out market analyses of palliative care for children (persons under 15 years of age).

5 A review of such methods can be found, e.g., in [35].
